# Author Correction: Structural basis for directional chitin biosynthesis

**DOI:** 10.1038/s41586-023-06553-z

**Published:** 2023-08-25

**Authors:** Wei Chen, Peng Cao, Yuansheng Liu, Ailing Yu, Dong Wang, Lei Chen, Rajamanikandan Sundarraj, Zhiguang Yuchi, Yong Gong, Hans Merzendorfer, Qing Yang

**Affiliations:** 1 State Key Laboratory for Biology of Plant Diseases and Insect Pests, Institute of Plant Protection, Chinese Academy of Agricultural Sciences, Beijing, China; 2 Shenzhen Branch, Guangdong Laboratory of Lingnan Modern Agriculture, Genome Analysis Laboratory of the Ministry of Agriculture and Rural Affairs, Agricultural Genomics Institute at Shenzhen, Chinese Academy of Agricultural Sciences, Shenzhen, China; 3 Faculty of Environment and Life, Beijing University of Technology, Beijing, China; 4 School of Bioengineering, Dalian University of Technology, Dalian, China; 5 Tianjin Key Laboratory for Modern Drug Delivery and High-Efficiency, Collaborative Innovation Center of Chemical Science and Engineering, School of Pharmaceutical Science and Technology, Tianjin University, Tianjin, China; 6 Center for Multi-disciplinary Research, Institute of High Energy Physics, Chinese Academy of Sciences, Beijing, China; 7 Department of Chemistry and Biology, School of Science and Technology, University of Siegen, Siegen, Germany

**Keywords:** Cryoelectron microscopy, Transferases

Correction to: *Nature* 10.1038/s41586-022-05244-5 Published online 21 September 2022

In the version of the article originally published, the chemical structure in Fig. 4a was missing a nitrogen symbol on the far right, which is now been inserted in the HTML and PDF versions of the article.

