# Supplementary material for: Structural basis for directional chitin biosynthesis
Source: Nature. 2022 Sep 21;610(7931):402–8. doi: 10.1038/s41586-022-05244-5 (PMC9556331; doi:10.1038/s41586-022-05244-5)
Supplement: Supplementary file 1 — This file contains Supplementary Fig. 1 and legends for Supplementary Videos 1 and 2. [file 41586_2022_5244_MOESM1_ESM.pdf]

nature portfolio

https://doi.org/10.1038/s41586-022-05244-5

Supplementary information

Structural basis for directional chitin biosynthesis

In the format provided by the authors and unedited

SI Guide

Structural basis for directional chitin biosynthesis

Wei Chen[1,2,8], Peng Cao[3,8], Yuansheng Liu[4,8], Ailing Yu[2], Dong Wang[4], Lei Chen[2], Rajamanikandan Sundarraj[5], Zhiguang Yuchi[5], Yong Gong[6,*], Hans Merzendorfer[7], Qing Yang[1,2,3,*]

Affiliations:

[1]State Key Laboratory for Biology of Plant Diseases and Insect Pests, Institute of Plant Protection, Chinese Academy of Agricultural Sciences, Beijing, China

[2]Shenzhen Branch, Guangdong Laboratory of Lingnan Modern Agriculture, Genome Analysis Laboratory of the Ministry of Agriculture and Rural Affairs, Agricultural Genomics Institute at Shenzhen, Chinese Academy of Agricultural Sciences, Shenzhen, China

[3]Faculty of Environment and Life, Beijing University of Technology, Beijing, China

[4]School of Bioengineering, Dalian University of Technology, Dalian, China

[5]Tianjin Key Laboratory for Modern Drug Delivery & High-Efficiency, Collaborative Innovation Center of Chemical Science and Engineering, School of Pharmaceutical Science and Technology, Tianjin University, Tianjin, China

[6]Center for Multi-disciplinary Research, Institute of High Energy Physics, Chinese Academy of Sciences, Beijing, China

[7]Department of Chemistry and Biology, School of Science and Technology, University of Siegen, Siegen, Germany

[8]These authors contributed equally: Wei Chen, Peng Cao, Yuansheng Liu

*Corresponding authors: qingyang@caas.cn (Q. Y.); yonggong@ihep.ac.cn (Y. G.)

<u>Table of Content:</u>

SI Figure 1 | Uncropped SDS-PAGE and western blot images from Extended Data Figures. **a**, SDS–PAGE analysis of purified *Ps*Chs1 in Extended Data Fig. 2a. **b**, Western blot analysis of *Ps*Chs1 truncation in Extended Data Fig. 2l. **c**, Western blot analysis of *Ps*Chs1 mutants in Extended Data Fig. 2j.

SI Video 1 | MD simulations of the apo *Ps*Chs1. The VLPGA loop adopts a closed conformation throughout the 50 ns simulation, which prevents the water from permeating freely through the channel.

SI Video 2 | MD simulations of the UDP/$(GlcNAc)_3$-bound *Ps*Chs1. UDP and $(GlcNAc)_3$ were removed from the complex structure before the simulation. The VLPGA loop adopts an open conformation throughout the 50 ns simulation, which allows the water to permeate freely through the channel.

SI Figure 1

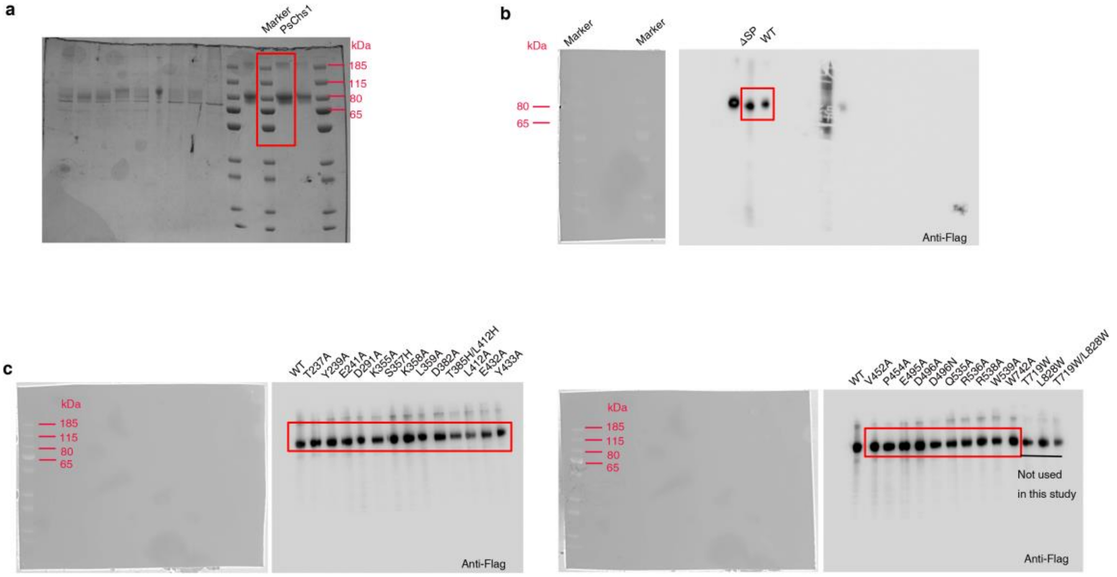
a
Marker
PsChs1
kDa
185
115
80
65
b
kDa
Marker
Marker
80
65
ΔSP
WT
Anti-Flag
c
kDa
185
115
80
65
WT
T237A
Y239A
E241A
D291A
K355A
S357H
K358A
L359A
D382A
T385H/L412H
L412A
E432A
Y433A
Anti-Flag
kDa
185
115
80
65
WT
V452A
P454A
E495A
D496A
D496N
Q535A
R536A
R538A
W539A
W742A
T719W
L828W
T719W/L828W
Not used
in this study
Anti-Flag
